# Rapid standardized operating rooms (RAPSTOR) in thyroid and parathyroid surgery

**DOI:** 10.1186/s40463-021-00525-x

**Published:** 2021-07-08

**Authors:** Hannah Ernst, Leigh Sowerby, Axel Sahovaler, Danielle Macneil, Anthony Nichols, John Yoo, Richard Hilsden, Julie Strychowsky, Kevin Fung

**Affiliations:** 1 Department of Otolaryngology—Head & Neck Surgery, Western University, London, Ontario Canada; 2 Department of Head and Neck Surgery Unit, General Surgery Department, Italian Hospital of Buenos Aires, Buenos Aires, Argentina; 3 Department of Otolaryngology- Head and Neck Surgery and Surgical Oncology, Sunnybrook Odette Cancer Centre, University of Toronto, Toronto, Ontario Canada; 4 Department of Surgery, Division of General Surgery, Western University, London, Ontario Canada

**Keywords:** Endocrine, Thyroidectomy, Parathyroidectomy, Efficiency, Cost minimization, Consecutive case scheduling

## Abstract

**Objective:**

To evaluate the impact of a high efficiency rapid standardized OR (RAPSTOR) for hemithyroid/parathyroid surgery using standardized equipment sets (SES) and consecutive case scheduling (CCS) on turnover times (TOT), average case volumes, patient outcomes, hospital costs and OR efficiency/stress.

**Methods:**

Patients requiring hemithyroidectomy (primary or completion) or unilateral parathyroidectomy in a single surgeon’s practice were scheduled consecutively with SES. Retrospective control groups were classified as sequential (CS) or non-sequential (CNS). A survey regarding OR efficiency/stress was administered. Phenomenography and descriptive statistics were conducted for time points, cost and patient outcome variables. Hospital cost minimization analysis was performed.

**Results:**

The mean TOT of RAPSTOR procedures (16 min; *n* = 27) was not significantly different than CS (14 min, *n* = 14) or CNS (17 min, *n* = 6). Mean case number per hour was significantly increased in RAPSTOR (1.2) compared to both CS (0.9; *p* < 0.05) and CNS (0.7; p < 0.05). Average operative time was significantly reduced in RAPSTOR (32 min; *n* = 28) compared to CNS (48 min; *p* < 0.05) but not CS (33 min; *p* = 0.06). Time to discharge was reduced in RAPSTOR (595 min) compared to CNS (1210 min, p < 0.05). There was no difference in complication rate between all groups (*p* = 0.27). Survey responses suggested improved efficiency, teamwork and workflow. Furthermore, there is associated decrease in direct operative costs for RAPSTOR vs. CS.

**Conclusion:**

A high efficiency standardized OR for hemithyroid and parathyroid surgery using SES and CCS is associated with improved efficiency and, in this study, led to increased capacity at reduced cost without compromising patient safety.

**Level of evidence:**

Level 2.

**Graphical abstract:**

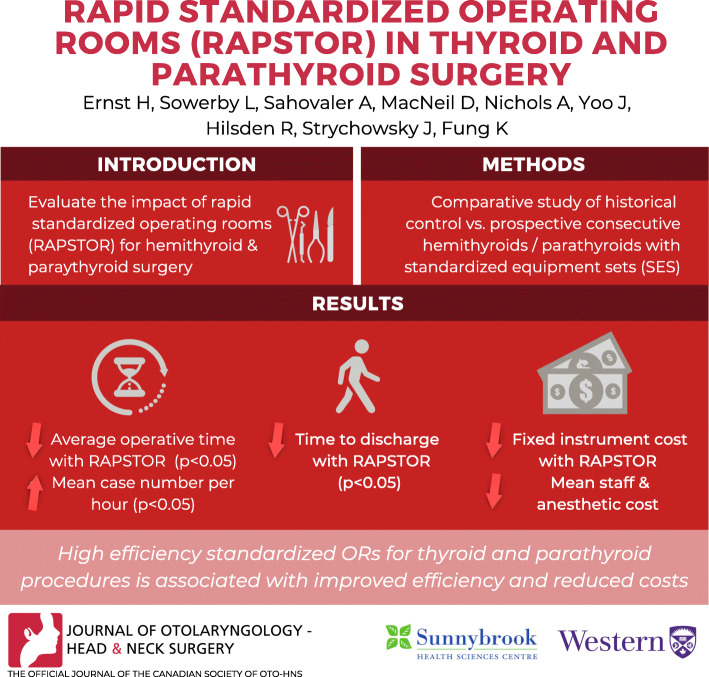

**Supplementary Information:**

The online version contains supplementary material available at 10.1186/s40463-021-00525-x.

## Introduction

Healthcare costs in Canada are expected to continue to increase annually. In 2016, an 11.1% of the gross domestic product was dedicated to healthcare, with hospitals consuming the largest component of funding (29.5%) [1]. Restricted funding in the context of increasing financial demand, requires that hospitals optimize the most resource intensive activities, such as operating room-related costs.

Many strategies, including the Lean and Six sigma (LSS) methodologies, have been adapted from the manufacturing industry to minimize process variability and the removal of non-value added procedures ultimately to improve productivity, personnel costs, reduced waste and financial performance [2]. Similarly, in the healthcare system, efficiency is highly prioritized. Efficiency in this setting refers to the useful application of both time and personnel addressing patient needs. In 2011, Cima et al. utilized LSS to characterize particular barriers to operative flow including, unplanned surgical volume variations, inefficient preoperative processes, excessive non-operative time, redundant information collection and lack of employee engagement [3]. Overall, there is a growing body of evidence regarding interventions that may improve overall OR efficiency. For example, standardized procedures and scheduling of consecutive surgeries decrease operative times [3]. Standardized equipment sets (SES), appear to reduce operative costs without extending OR time or compromising safety [4, 5]. Involving staff that are familiar with the daily procedures and consecutive case scheduling (CCS) have demonstrated reduced patient preparation time, leading to overall reduction in procedure duration [6–8]. Importantly, improvements which have a specific multidisciplinary focus appear to have a greater capacity to increase surgical efficiency, and also result in a favorable impact on staff morale [9].

Within the field of Otolaryngology – Head and Neck Surgery, thyroid and parathyroid related surgery is commonly performed and therefore, it is imperative to optimize OR efficiency and minimize extraneous costs. In 2016, Mascarella et al. demonstrated that eliminating non-value added components, such as indiscriminate case scheduling in parathyroid and thyroid surgery, could increase daily case volume and decrease (TOT) turnover time without increasing perioperative complications [10]. To date; however, no study evaluating thyroid and parathyroid surgery OR-related efficiency has encompassed the broad spectrum of outcomes: from OR time points, patient quality and safety, OR environment and treatment costs. It is our aim to develop a rapid standardized OR (RAPSTOR) through SES and CCS and to subsequently evaluate the impact upon intra-operative and post-operative time points, patient safety parameters, staff perception of efficiency and hospital-based costs.

## Methods

### Study design

Comparative study including a prospective cohort and a historical control.

### Setting

A single otolaryngologist’s practice at a tertiary care teaching hospital.

Non-identifying patient health information was collected on the day of surgery as well as retrospectively through the electronic medical record. Time intervals were calculated based on perioperative tracking reports. Additional information regarding hospital related costs were provided by Decisoin Support Services.

### Development of standardized equipment sets

This was achieved through extensive discussion between the primary surgeon in consultation with other head and neck surgeons at the same institution. The goal was to create a surgical tray with a minimum number of instruments required for thyroid and parathyroid surgery. In total, 40 instruments were removed from the standard thyroid/parathyroid equipment tray to create the SES.

#### Patient groups

All hemithyroidectomies, completion thyroidectomies and unilateral parathyroidectomies were included from a total of 5 operative days. Patient demographics were collected including age, surgical indication (pathology, primary hyperparathyroidism), date of OR, length of hospital stay, co-morbidities, ASA class and perioperative complications (hematoma, hypocalcemia, vocal cord paralysis).

##### RAPSTOR group

Patients booked for unilateral thyroid or parathyroid surgery that were classified as ASA 1 or 2 were scheduled consecutively. A unilateral parathyroidectomy approach was achieved by the use of localizing pre-operative sesamibi parathyroid scans. Furthermore, patients were enrolled based on their availability for a particular surgical day and place on the current waiting list. These were collected over 5 separate operative days from May 2017 to February 2018. The frequency and dates of RAPSTOR operative days were determined by operating room administration as it depends largely on the availability of operative and perioperative staff.

##### Comparison group

Matched patients (booked for unilateral thyroid or parathyroid surgery with ASA 1 or 2) were identified retrospectively Time intervals began at the closest identifiable date to the RAPSTOR start and varied in length in order to capture the appropriate ASA and procedure matched patients in the correct scheduling paradigm. They were grouped as either,

- Consecutive (CS): at least 3 consecutively scheduled cases. These were collected from 5 separate operative days from July 2016 until November 2017. A larger time interval was required, as similar ASA and procedure patients were not routinely scheduled consecutively.
- Non-consecutive (CNS): less than or equal to 2 consecutively scheduled cases. Patients were identified from 5 operative days from January 2017 until April 2017.

#### Time intervals

**Fig. 1:**
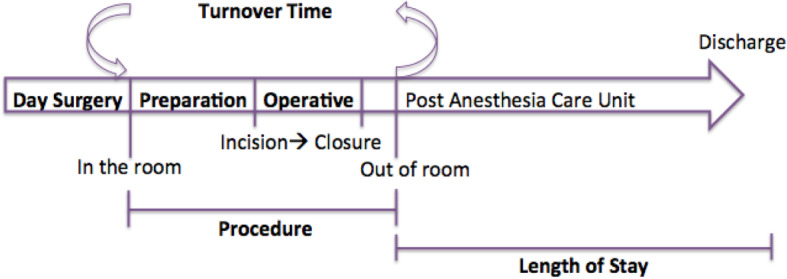
Flow of time intervals through the operative day

Time points (Fig. 1) for RAPSTOR patients were collected prospectively. CS and CNS time points were collected retrospectively through individual patient peri-operative chart evaluation. If complete data were unable to be located, which was required for a specific time interval, that patient would be excluded from that specific time interval calculation. Descriptive statistics and unpaired student t-test was utilized for analysis between groups. Of note, at our institution intraoperative parathyroid hormone is not available and thus, time to request and wait for frozen section pathology was included in the parathyroid calculations.

#### Patient quality and safety

Information regarding intra-operative and perioperative information was collected for RAPSTOR (prospectively) and non-standardized (retrospectively) patients through patient electronic record review. Specifically, hematoma, hypocalcaemia, vocal cord immobility and co-morbidity exasperation were considered. Hematoma was defined as a collection requiring drainage in the operating room. Hypocalcaemia was defined as objective drop in calcium requiring either intravenous and/or oral supplementation. Suspicion of vocal cord immobility in the post-operative period is based on voice or swallowing symptoms. If present, a flexible nasopharyngoscopy examination is standard at our institution. Descriptive statistics and unpaired student t-test was utilized to evaluate RAPSTOR vs. non-standardized patient differences.

#### Operating room environment

A survey (Additional file 1: Appendix 1) was constructed prior to the initiation of RAPSTOR in collaboration with the primary surgeon and OR charge nurse. It was distributed, in a paper format, to health professionals (Nursing, anesthesia, staff surgeon and residents) post- RAPSTOR OR for evaluation of OR perceptions related to workflow efficiency and perceived stress level. Descriptive statistics and ANOVA analysis was utilized to elucidate differences between disciplines.

#### Operating room staff

At our institution, it is standard for operating room nurses to circulate through a wide number of specialties and gain a wide spectrum of skills. It is however, common for an operating room nurse to narrow their focus to a select few after training, but not mandatory. There is therefore a spectrum of experience among Otolaryngology operating nurses at our institution. Non-RAPSTOR room nursing staff was selected by OR administrators. RAPSTOR nursing staff was selected in collaboration with the primary surgeon to maximize degree of experience. This did not include peri-operative care such as in the surgical day unit or PACU. Both Anesthesia and Otolaryngology residents participated in RAPSTOR and non-RAPSTOR rooms.

#### Financial analysis

A hospital based cost analysis was conducted comparing RAPSTOR cases vs CS. Non-consecutively scheduled comparisons were excluded secondary to limited peri-operative information available in the electronic record. Furthermore, the differentiating factor between both groups would be the SES rather than both CCS and SES. All costs related to intra-operative, post anesthesia care unit and hospital stay were included (Table 2).

- *Registered nurse, registered practical nurse, OR aide hourly wage* was included provided by London Health Sciences Center Department of Decision Support. Mean staff cost per procedure hour was calculated and descriptive statistics were utilized including, mean staff cost per OR hour. See Additional file 2: Appendix 2.
- *Anesthesia-related costs* were adapted from work by Hilsden et al., adjusted for mean procedure length and total number of cases. See Additional file 2: Appendix 2.
- *Instrument related costs* were adapted from Chin et al (2014) and John-Baptiste et al (2016). See Additional file 2: Appendix 2.
- Peri-operative cost was calculated in conjunction with Decision Support Services at London Health Sciences Centre. Surgical costs (cost per minute of workload, supplies used) and direct materials cost was included encompassing intra-operative, PACU and post-operative hospital stay. See Additional file 2: Appendix 2.

All statistical analysis was conducted through Prism 7.02017 Statistical program.

## Results

### Patient demographics

Mean patient age in RAPSTOR (52.9 ± 3.1, *n* = 29), CS (51.1 ± 2.7, *n* = 27) and CNS (58.43 ± 1.863, *n* = 7) were not statistically different (ANOVA; *p* > 0.05). Baseline co-morbidities are listed in Table 1. Similar number of procedures were performed in all groups with hemithyroidectomy being most common (Table 2). Pathology characteristics are listed in Table 2.

**Table 1:** Patient Demographics and Co-morbidities

Patient Demographics	RAPSTOR	CS	CNS
Mean Age ± SD	52.93 ± 3.074, *n* = 29	51.11 ± 2.667, *n* = 27	58.43 ± 1.863, *n* = 7
**Cardiovascular**			
Cardiac Arrhythmia	4	0	0
Hypertension	8	0	1
Cerebrovascular Event	2	0	0
**Respiratory**			
Smoking (Active or Previous)	3	2	3
Respiratory Disease	1	1	0
**Endocrinopathies**			
Hypothyroidism	2	3	4
Hyperthyroidism	0	0	1
Primary Hyperparathyroidism	2	2	3
Diabetes Mellitus	10	0	1

**Table 2:** Thyroid and Parathyroid Pathology

		RAPSTOR	CS	CNS
Thyroid lesion size	≤ 1 cm	3	4	0
	> 1 cm but ≤2 cm	5	10	0
	> 2 cm but ≤4 cm	9	6	1
	> 4 cm	1	2	2
Parathyroid size	≤ 1000 mg	5	3	2
	> 1000 mg	5	1	1

### Time intervals

Turnover times between all groups were not statistically different, between groups (Table 3; Fig. 1) including on subgroup analysis. Operative time for RAPSTOR (32 ± 12 min) was decreased compared to CNS (48 ± 16 min; *p* < 0.05) but not to CS (38 ± 10 min; *p* > 0.05) (Table 4). Subgroup analysis revealed reduced operative time in RAPSTOR hemi-thyroidectomies (28 ± 6 min) compared to both CS (37 ± 8 min *p* < 0.001) and CNS (46 ± 7 min; *p* < 0.05). Procedure time, which combines both preparation, surgical time and waiting for PACU transfer, was significantly lower between RAPSTOR (51 ± 14 min) and CS (60 ± 13 min, *p* < 0.05) and CNS (77 ± 19 min, *p* < 0.0001). Specifically, for hemi-thyroidectomies, the procedure time was markedly reduced in the RAPSTOR cohort (43 ± 6 min) compared to CS (61 ± 13 min, *p* < 0.0001) and CNS (72 ± 19 min, *p* < 0.0001). Furthermore, CS procedure and surgical time was significantly shorter compared to CNS (*p* < 0.05).

**Table 3:** Turnover Time

	RAPSTOR ± SD	CS ± SD	*P* Value	CNS ± SD	*P* value
All	16 ± 7.6 (n = 27)	14.3 ± 6.4 (*n* = 14)	0.47	16.8 ± 4.0 (*n* = 6)	0.80
Thyroid	15.2 ± 7.7 (*n* = 17)	14.9 ± 6.0 (*n* = 10)	0.91	17.8 ± 4.1 (*n* = 4)	0.54
Parathyroid	18.9 ± 1.7 (n = 10)	16.5 ± 1.5 (*n* = 4)	0.41	15 ± 4.2 (n = 23)	0.17

**Table 4:** Operative Time

	RAPSTOR ± SD	CS ± SD	*P* Value	CNS ± SD	*P* value
All	31.5 ± 12.5 (*n* = 28)	37.5 ± 10.0 (*n* = 27)	0.062	48.1 ± 16.1 (*n* = 7)	0.0052
Thyroid	27.8 ± 5.8 (*n* = 19)	36.8 ± 8.3 (*n* = 23)	0.0003	46 ± 7.3 (*n* = 4)	0.0031
Parathyroid	42.7 ± 12.3 (*n* = 9)	40 ± 18.8 (n = 4)	0.76	51 ± 12.1 (*n* = 3)	0.41

### Mean cases per hour

**Fig. 2:**
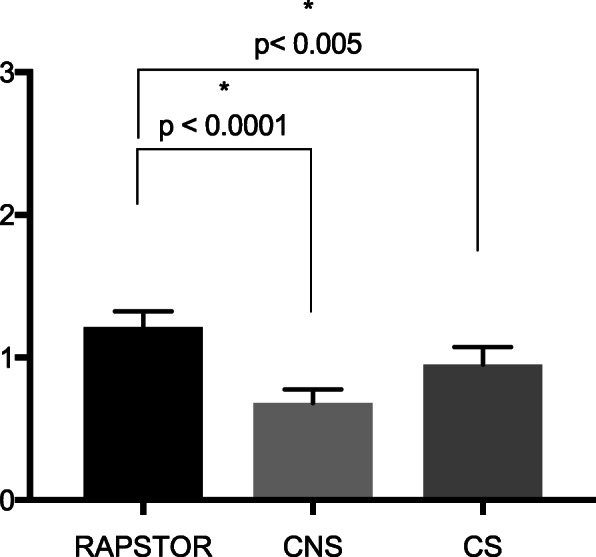
Number of cases per hour in RAPSTOR as well as consecutive (CS) and non-consecutive (CNS) operative days

Mean number of cases per hour (Fig. 2) was increased in RASPTOR (1.2 ± 0.05) compared to both CS (0.9 ± 0.04; *p* < 0.01) and CNS (0.7 ± 0.04; *p* < 0.0001).

### Patient quality and safety

Though hypocalcemia was the most common complication, there was no significant difference (*p* > 0.05) in complication frequencies in RAPSTOR groups when compared to CNS and CS groups. No hematoma or vocal cord immobility was detected in either group. Length of hospital stays in RAPSTOR group were significantly shorter (595 ± 102 min) than the CNS group (1210 ± 298 min; p < 0.05).

### Operating room environment and perceptions

Overall, the perception of a RAPSTOR operative day experience for staff was perceived to be improved compared to the traditional operative set-up and flow (Table 5). Operating room perceived efficiency improved while not concurrently increasing the perceived level of environmental stress by the staff. This impression was similar across all disciplines sampled.

**Table 5:** RAPSTOR OR Environment Perceptions

	Nurse	Resident	Anesthesia	Mean Total score	*P* Value
General	2.4 (n = 7)	2.8 (n = 4)	2.6 (n = 3)	2.6 (n = 14)	NS (*p* = 0.83)
Efficiency	3.4 (n = 7)	3.0 (n = 4)	3.3 (n = 3)	3.3 (n = 14)	NS (*p* = 0.67)
Stress	2.4 (*n* = 8)	2.0 (n = 4)	2.0 (n = 3)	2.1 (n = 15)	NS (*p* = 0.80)

Much worse: 0; somewhat worse 1; the same 2; somewhat better 3; much better 4

### Cost analysis

There was an overall cost reduction in RAPSTOR compared to CS cases across all cost parameters (Table 6). The fixed instrument cost per case was reduced by 45%. Time sensitive costs, such as mean staff cost and anesthetic-related cost were both decreased (1.7 and 18%, respectively).

**Table 6:** Cost Parameters

Cost Parameter	RAPSTOR ($CAD)	CS ($CAD)
Mean staff (cost/hr./case)	220.36 (*n* = 28)	224.12 (*n* = 14)
Instrument (cost/case)	9.45	17.20
Mean Anesthesia (cost/hr./case)	6.08 (*n* = 15)	7.43 (*n* = 27)
Post anesthesia Care Unit (mean cost/patient)	669 (*n* = 19)	723 (*n* = 11)

## Discussion

In a healthcare system that is increasingly constrained financially, it is imperative to optimize surgical resources and minimize cost. Our study is the first to evaluate hospital based costs and to demonstrate minimization with respect to OR efficacy, patient quality and safety as well as how these changes impact the perceived operative environment.

### Operating room utilization

We have demonstrated a significant improvement in operative time, total procedure time, and overall case number per hour in consecutively scheduled similar cases, particularly hemithyroidectomies. This suggests a relationship between surgical team procedure familiarity and operative efficiency. Henaux et al, reported that an inverse relationship was observed between operative workflow disruptions and the degree of familiarity of the surgeon and surgical nursing staff (2019) [11]. Surgeon technique and equipment preferences become known and anticipated, thereby minimizing equipment and technical disruptions. Familiarity in our setting was cultivated by utilizing consecutive scheduling of similar patients, specific nursing staff and standardized surgical set-ups within a single surgeons’ practice. Parathyroidectomy operative time is likely constrained because of the need to wait for pathology intraoperatively. This is an extrinsic factor that is not within the control of the surgical team. Upon evaluating those specific time points in the future, additional efficiencies may be identified.

Turnover times in the RAPSTOR group were surprisingly not decreased compared to CS and CNS. Stepniak et al. demonstrated decreased turnover times with a fixed OR team and consecutively scheduled similar cases (inguinal hernia repair and laparoscopic cholecystectomies) (2017) [6]. Importantly, their staff assignments did not vary through the day. Though our study worked with particular operative staff, including familiar nurses, with the advent of scheduled breaks and the need for OR aides to assist multiple rooms at once, the number of staff present was not always consistent. Similarly, Bhatt et al. demonstrated decreased turnover times when evaluating a myriad of dissimilar cases (2014) [12]. Tremendous difference; however, can be seen regarding the structure of turnover such as the implementation of parallel processing which utilizes a rigorous morning checklist and employed ‘core technician’ [12]. Their primary responsibility was liaising with the sterile processing core regarding cart priorities as well as ensuring that case carts were outside the appropriate operating room. This naturally allowed the nursing staff to focus on additional priorities such as patient readiness. Perhaps these strategies could be applied to our institution in future quality improvement assessments to improve this time interval and better match the landscape of current literature.

From an educational perspective, an advantage may be found in allowing resident-surgeons to participate in consecutively scheduled cases with a single surgeon. At our center, RAPSTOR cases were performed by all training levels of otolaryngology residents under the direct supervision of a experienced staff surgeon. The improvement of operative time with the inclusion of trainees is consistent with literature that suggests that repetition of procedures improves surgical time and technique [13, 14]. For example, in vascular and robotic assisted surgery, resident surgeons exposed to consecutively scheduled similar cases, under the coaching of a single surgeon, had the opportunity to not only gain operative experience, but to improve familiarity and sharpen technique through the operative day [13]. Maruthapu et al. demonstrated that not only does staff surgeon and resident surgeon cumulative experience positively impact operative time, but the number of collaborations between specific consultant-surgeons and specific trainees (2016) [15].

Improving cases per hour has a potential multidimensional impact. Thyroid and parathyroid surgery are amongst the most common procedures performed in Otolaryngology. As a result, the waiting list of patients is large. Increasing the number of cases performed each operative hour, would open up potential time for additional patients and improve the waiting lists. Mascerella et al demonstrated similar findings with the development of a high efficiency protocol for endocrine surgery (2016) [10]. Perhaps this model could be applied to other common otolaryngology procedures, such as adenoidectomy or functional endoscopic sinus surgery, to improve operating room efficiencies and decrease wait times.

### Patient safety

While these variables are of benefit, it is important to also acknowledge that these improvements should not come at the expense of patient safety and surgical quality. In our study, decreased operative time translates to decreased anesthetic exposure and potentially improved wait times. RAPSTOR cases compared to non-consecutively scheduled cases had significantly decreased post-operative hospital courses and no significant increase in perioperative complications. Improved post-operative stay in our setting is likely multifactorial; however, it is possible that PACU factors such as staffing demand, familiarity with the cases and motivation for efficiency played an important role. The significant difference between PACU time to discharge found in RAPSTOR vs both CS and CNS groups without a significant difference between the CS and CNS may reflect these factors. Such factors were not specifically evaluated in the PACU in the current study but certainly would be an interesting future direction. Similar findings have been described through high efficiency endocrine operating protocols [10] . Appropriate and prompt monitoring done in the post anesthetic care unit was initiated for all parathyroidectomies and completion thyroidectomies. This allowed for titration of supplementary vitamin D and calcium prior to discharge, if required. This is standard practice at our institution as rapid intraoperative parathyroid hormone is not available. With the advent of such a test, not only could operative time be further minimized (no requirement for pathology), but it would allow for a more rapid predictor of post-operative hypo-parathyroidism [16].

### Perception of operating room environment

Improving operating room efficiency did not come at the expense of operating room perceived stress. To our knowledge, stress perception has not been evaluated in this context before. Though our strategies at improving efficiency were different, LEAN management techniques applied at a Michigan academic institution to optimize surgical efficiency, similarly showed an improvement in teamwork and morale [9].

### Hospital-based cost

Through equipment minimization, and consecutive similar case scheduling, overall cost reduction is possible. Intuitively, equipment costs, including processing and depreciation, improve with minimizing the equipment required per case. Importantly, however, improving operative time means less anesthetic-related expenses and overall staff cost (less overtime required). Shorter post-operative hospital course also translates into less cumulative hospital non-operative expenses such as ward nursing, medications, and nourishment. Furthermore, as the number of available hospital beds per 1000 people in Canada decreased from 3.0 in 2006 to 2.6 in 2016, it is imperative to minimize in-patient stay particularly if it has been shown to be equivocal from a safety perspective [1].

### Limitations

This study was limited by the small sample size and inclusion of a single surgeons’ practice. While this may eliminate surgeon-dependent variables between patients– such as as variation in experience, operative time and the role of learners – such variables may vary widely between Otolaryngologists and limit generalizability regarding the degree to which improvements may occur. Furthermore, the use of a retrospective comparison group introduces challenges related to the electronic medical record and amount of available patient information available (for example, co-morbidities and course in hospital). Future initiatives could potentially include the entire Otolaryngology- Head and Neck Surgery department to better evaluate how such changes impact departmental outcomes, rather than a single member’s practice and include a prospective comparison group. Furthermore, it is plausible that Hawthorne effect may have been experienced by OR staff which were aware that a RAPSTOR CS room was scheduled for the day. The number of cases booked and requirement for efficiency to complete the case load could prompt a change in motivation for efficiency throughout the day. This; however, is not viewed by the author as diminishing the positive impact that this possible change in motivation had on time points. It may actually serve to highlight an important method of motivating an operative team such as daily discussion regarding case-load and need for optimizing modifiable delays. Additionally, there was limited peri-operative data available for non-RAPSTOR cases prior to 2017 as time preparing patients in day surgery, delay in transfer to operating room and patients arriving to day surgery late. All have the potential to shed light on areas of potential delay in the peri-operative setting. Our survey constructed to evaluate the operating room environment was not validated. A possible future study could include validation of this questionnaire for more widespread use in the surgical community. Furthermore, inclusion of both hemi-thyroidectomy and completion thyroidectomy can obscure post-operative time points such as length of stay. Completion thyroidectomies have an increased risk of hypocalcaemia and thus, appropriate blood work and symptom monitoring is required. As our institution, General Surgery and now, Otolaryngology- Head and Neck Surgery have adopted RAPSTOR models for a portion of operative days.. The number of dedicated days may increase as additional surgeons are included. Future directions may include a similar prospective study but encompassing additional subspecialties or procedures (for example, microlaryngoscopy).

## Conclusion

Rapid standardized operating rooms, with consecutively scheduled, similar cases and equipment minimization, improve operating room efficiency in parathyroid and hemi-thyroidectomy surgery. The impact is multifaceted involving time (operative, patient waiting lists, post-operative course) as well as cost (operative and non-operative), optimizing healthcare resources at our institution without sacrificing patient safety and surgical quality.

## Supplementary Information

**Additional file 1.**

**Additional file 2.**

## Data Availability

Upon request of the corresponding author.
