# Supplementary material for: Rapid standardized operating rooms (RAPSTOR) in thyroid and parathyroid surgery
Source: J Otolaryngol Head Neck Surg. 2021 Jul 8;50:44. doi: 10.1186/s40463-021-00525-x (PMC8265141; doi:10.1186/s40463-021-00525-x)
Supplement: Supplementary file 1 — Additional file 1. [file 40463_2021_525_MOESM1_ESM.docx]

Appendix 1

Questionnaire for operating room staff:

0 much worse

1 somewhat worse

2 the same

3 somewhat better

4 much better

Question #1: How did this OR compare with traditional?

Question #2: How did the efficiency of this OR compare with traditional?

Question #3: How was your level of stress compared to traditional?
