# Supplementary material for: Rapid standardized operating rooms (RAPSTOR) in thyroid and parathyroid surgery
Source: J Otolaryngol Head Neck Surg. 2021 Jul 8;50:44. doi: 10.1186/s40463-021-00525-x (PMC8265141; doi:10.1186/s40463-021-00525-x)
Supplement: Supplementary file 2 — Additional file 2. [file 40463_2021_525_MOESM2_ESM.docx]

Appendix 2

- *Registered nurse, registered practical nurse, OR aide hourly wage*

**Step 1:**

Total staff cost/hr = (mean no. RN)(contract wage $/hr) +(mean no. OR aide) (contract wage $/hr) + (mean no. RPN)(contract wage $/hr)

**Step 2:**

Staff cost/OR hour= (step 1)(total procedure length +TOT)/total no. cases

- *Anesthesia-related costs* **Step 1:**

((O2 flow)*(% Agent /100)) / Vapor (mL)/ Liquid (mL)

= ((1500mL vapour/min)(2.5%/100)) /(183mL vapour/ liquid mL)

= 0.205 liquid mL/min

**Step 2:**

(Answer Step 1)*((cost bulk agent ($)/ bottle size (mL))

= 0.205 liquid mL/min* (($146.83/250mL liquid))

= (0.205 liquid mL/min*($0.587/mL liquid)

= $0.1204/min

**Step 3:**

(Step 2)*(60 minutes)*(total hours done of **surgical procedure** / no. cases)

= ($0.1204/min)(60minutes)(total hours done of surgical procedure/no.cases)

= ($7.224/hr)(total hours done of surgical procedure/no.cases)

- *Instrument related costs*

Total cost_tray_ = (n_procedures_ * n_instruments_)((cost_time_ (time_decontaminat_e+time_package_) +(cost_depreciation_)

= (n_procedures_ * n_instruments_) (($0.006/s (4.02s +17.5s)/instrument) +($0.06/instrument)

=(n_procedures_ * n_instruments_)(($0.129/instrument) +($0.06/instrument))

= (n_procedures_ * n_instruments_) ($0.189/instrument)
